# A Chinese case of Nakajo–Nishimura syndrome with novel compound heterozygous mutations of the PSMB8 gene

**DOI:** 10.1186/s12881-020-01060-8

**Published:** 2020-06-08

**Authors:** Tao Jia, Yi Zheng, Cheng Feng, Tielin Yang, Songmei Geng

**Affiliations:** 1 Department of Dermatology, Northwest Hospital, The Second Hospital Affiliated to Xi’an Jiaotong University, Xi’an, China; 2 Key Laboratory of Biomedical Information Engineering of Ministry of Education, Biomedical Informatics & Genomics Center, School of Life Science and Technology, Xi’an Jiaotong University, Xi’an, Shaanxi People’s Republic of China

**Keywords:** Nakajo-Nishimura syndrome, *PSMB8*, Compound heterozygous mutations, PRAAS

## Abstract

**Background:**

Nakajo-Nishimura syndrome (NNS) is an autosomal recessive heredity disorder, one of a spectrum of autoinflammatory diseases named proteasome-associated autoinflammatory syndrome (PRAAS) caused by mutations of *PSMB8* gene. NNS is characterized by pernio-like skin rashes, intermittent fever, and long clubbed fingers and toes with joint contractures, partially with progressive lipomuscular atrophy, emaciation, hepatosplenomegaly and basal ganglion calcification.

**Case presentation:**

We presented a sporadic case of NNS with compound heterozygous mutations in the *PSMB8* gene. The 4-year-old boy was affected by progressive erythematous plaques on his nose and gradually involved hands and feet later with characteristic appearance of long clubbed fingers. The repetitive periodic intermittent fever was recorded. By gene sequencing, novel compound heterozygous mutations c.373C > T (p.R125C) and c.355G > A (p.D119N) in the *PSMB8* gene were found. The patient responded well to low dosage of oral methylprednisolone.

**Conclusions:**

We reported novel compound heterozygous mutations in *PSMB8* in a sporadic Chinese NNS patient.

## Background

Nakajo-Nishimura syndrome (NNS, OMIM#256040), an autosomal recessively inherited disorder, was originally reported by a Japanese scholar in 1939 [1]. Up to now, approximately 30 cases have been reported all over the world, mostly from Japan and only a few from China and Europe. Mutations in the *PSMB8* (*proteasome 20S subunit beta 8*, MIM 177046) gene, which encodes the β5i subunit of immunoproteasome, have been identified to cause NNS [2].

Patients with NNS show pernio-like skin rashes since infancy, and gradually develop partial lipodystrophy mainly in the face and upper extremities, as well as nodular erythema-like skin eruptions. Characteristic long clubbed fingers with contracture of the interphalangeal joints accompanied by remittent fever would also appear [3]. Other symptoms would gradually appear, such as progressive partial lipomuscular atrophy and emaciation, hepatosplenomegaly, and basal ganglia calcification [4].

Here, we presented a sporadic NNS Chinese case with novel compound heterozygous mutations in exon 3 of *PSMB8*.

## Case presentation

**Fig. 1:**
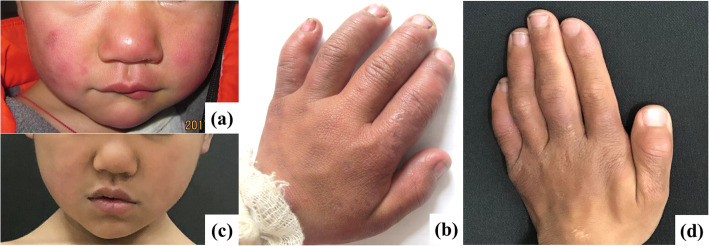
Clinical findings at first visit and after treatment. Multiple erythematous plaques on his face, hands and feet, long clubbed fingers before treatment **a, b**. Significant improvement after therapy **c, d**

The patient was a 4-year-old Chinese boy, presented with progressive erythematous plaques on his nose for 6 months. The skin lesions gradually involved all the hands and feet (Fig. 1), with an intermittent fever. No other symptoms like stomachache, or diarrhea were complained. His parents denied trauma or allergy history. No parental consanguinity or any remarkable family history was recorded.

On physical examination, lymph nodes in bilateral axillary moderately enlarged. No obvious abnormality of internal organs was found. Multiple erythematous plaques were noted on his face and extremities. He had clubbed fingers, toes and also had mild joint contracture in his fingers. Laboratory analysis revealed a slightly anemia with hemoglobin 107 g/L (normal 120-140 g/L). Serum aspartate aminotransferase (AST) 44 IU/L (normal 15-40 IU/L), lactic dehydrogenase (LDH) 347 IU/L (normal 120-250 IU/L), hydroxybutyrate deacidification enzyme (HBDH) 286 IU/L (normal 26-195 IU/L) were elevated, while erythrocyte sedimentation rate, ANA series tests and other routine tests were negative. X-ray of chest showed increased lung markings. Serial cranial CT scans were taken which revealed no basal ganglia calcification. Abdominal CT scan didn’t show hepatosplenomegaly.

**Fig. 2:**
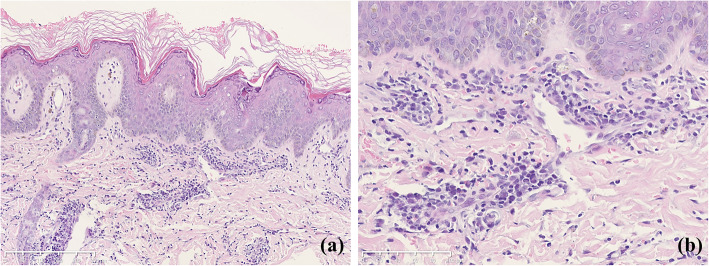
Biopsy results. H&E staining of erythematous lesions on hands. **a** Hyperkeratosis, irregular epidermal hyperplasia, mild spongy edema and single necrotic keratinocytes in epidermis along with mild vacuolar degeneration. Scale bars: 250 μm. **b** Medium monocytes with neutrophilic cells infiltrated around dermal vessels. Nuclear dust and mitotic figures were seen. Scale bars: 100 μm

Skin biopsy from the hand lesion showed hyperkeratosis, irregular epidermal hyperplasia, mild spongy edema and single necrotic keratinocytes in epidermis along with mild vacuolar degeneration. Medium monocytes with neutrophilic cells infiltrated around dermal vessels. Nuclear dust and mitotic figures were seen (Fig. 2a, b). Immunohistochemical examination showed that the most infiltrating cells were positive for CD3, CD68 and MPO.

**Fig. 3:**
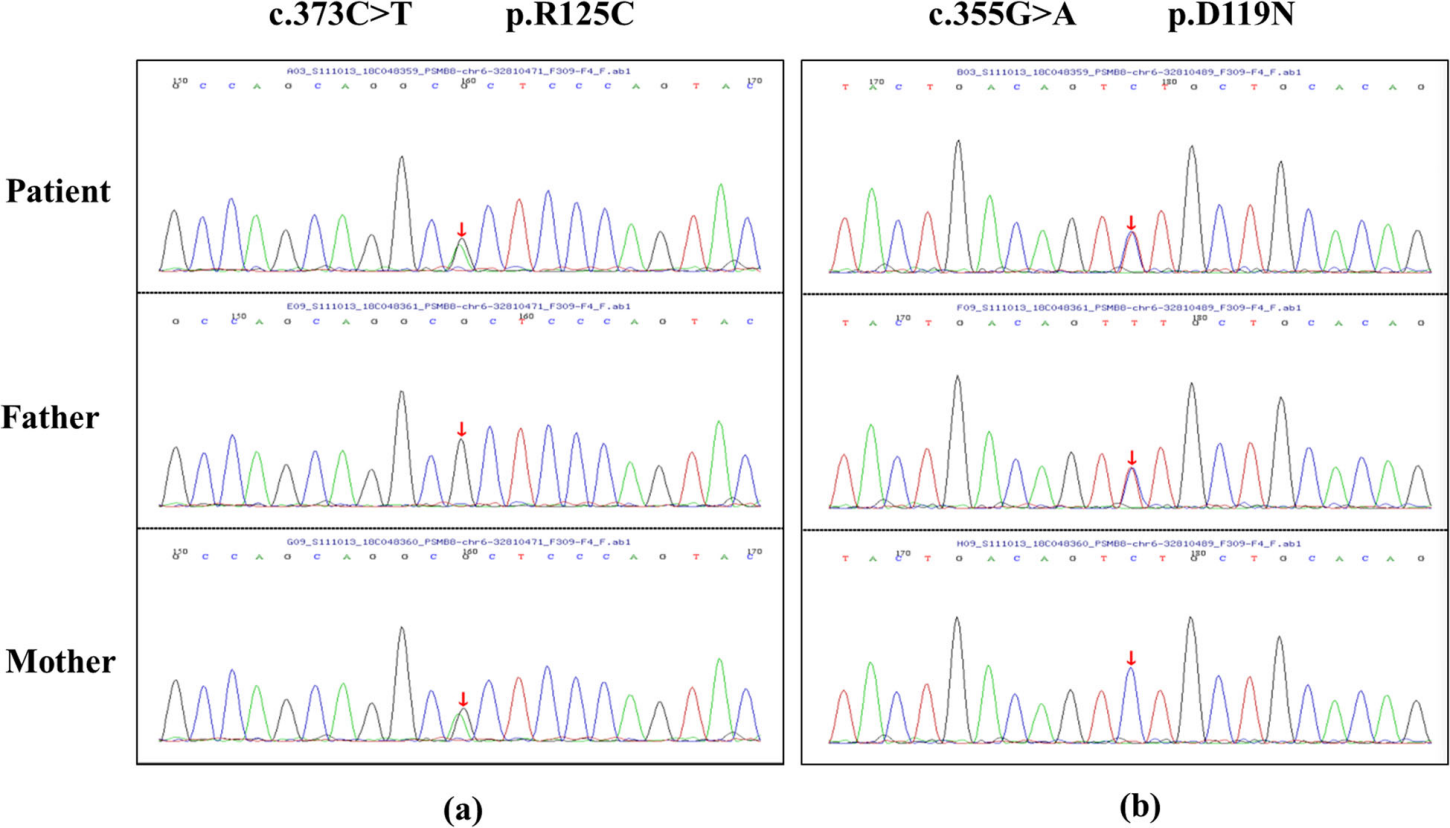
Gene sequencing results. Sanger screening revealed a heterozygous c.373C > T (p.R125C) mutation **a** and c.355G > A (p.D119N) mutation **b** in exon 3 of the *PSMB8* gene in the patient, a heterozygous c.373C > T (p.R125C) mutation in his mother and a c.355G > A (p.D119N) mutation in his father

Furthermore, we collected blood samples from the patient and his parents on receipt of informed consent. Whole exome sequencing was performed and results were revalidated by Sanger sequencing. The results showed compound heterozygous mutations c.373C > T (p.R125C) and c.355G > A (p.D119N) in the exon3 of the *PSMB8* (NM_004159.5; MIM 177046) gene (Fig. 3a, b) in proband. His parents were both heterozygous carriers with no clinical symptom. Based on the clinical manifestations and the genomic mutation on *PSMB8* gene, the diagnosis of Nakajo–Nishimura syndrome was made.

After the treatment with methylprednisolone 8 mg/d for 2 months, the lesions improved (Fig. 1) and the lymph nodes in bilateral axillary returned to normal size. The erythematous plaques on his face disappeared eventually with the dosage of methylprednisolone tapered.

## Discussion and conclusion

The diagnosis criteria of NNS consists of at least 5 of the 8 features including autosomal recessive inheritance (parental consanguinity and/or familial occurrence), pernio-like purplish rashes on hands and feet (appearing in winter since infancy), haunting nodular erythema with infiltration and induration (sometimes circumscribed), repetitive spiking fever (periodic, not necessarily), long clubbed fingers and toes with joint contractures, progressive partial lipomuscular atrophy and emaciation (marked in upper part of body), hepatosplenomegaly and basal ganglion calcification [5]. Our patient matched with 5 features, so the diagnosis of NNS was made, although lipomuscular atrophy and emaciation, hepatosplenomegaly or basal ganglion calcification weren’t found.

NNS belongs to a spectrum of autoinflammatory diseases named proteasome-associated autoinflammatory syndrome (PRAAS) caused by mutations of *PSMB8* gene. Mutations of *PSMB8* could cause JMP syndrome which characterized with joint contractures, muscle atrophy, microcytic anemia and panniculitis-induced lipodystrophy [3], CANDLE which characterized with chronic atypical neutrophilic dermatosis with lipodystrophy and elevated temperature [5], and JASL (Japanese autoinflammatory syndrome lipodystrophy) [6]. Because the similarities and overlapping symptoms among NNS, CANDLE, JPM, and JASL, it’s hard to discriminate these disease clearly [6]. Pernio-like rashes and repeated fever can always be observed in CANDLE and NNS. Long clubbed fingers are characteristic features for NNS patients. Joint contractures and muscle atrophy are prominent in JMP patients, while macroglossia and cardiac disease, which lead to death in middle age, usually occur in patients of JASL [4]. Hereby, long term following up of the patient is necessary to observe whether patient would develop from one of the PRAAS to another.

As both NNS and CANDLE are autosomal recessive diseases, most of the cases were caused by homozygous missense mutations in *PSMB8* gene. Nevertheless, heterozygous p.T75M mutation has been reported in CANDLE syndrome. And there was also a Hispanic PRAAS case presented compound heterozygous mutations p.T75M and p.A92T (a novel missense mutation) on *PSMB8* [7–10]. In our case, the two missense mutations (p.R125C and p.D119N) were absent in HGMD database, ClinVar database, ExAc database and 1000 Genomes database. However, in-silico analysis by Mutation Taster, Polyphen-2, REVEL, SIFT and Provean, both mutations were predicted as damaging. Based on the above reports, we believe that compound heterozygous mutations could be pathogenic to NNS. Our study extended the mutation spectrum and pattern of gene mutations of NNS.

Our case supposed to be the first NNS patient with compound heterozygous mutations in *PSMB8*. Whether patient with compound heterozygous mutations presented with mild symptoms, or different mutations impact variable clinical characteristics should be explored further based on more case reports and retrospective analysis.

Although the autoinflammatory symptoms such as pernio-like lesions and repetitive fever have improved markedly in our patient after treatment, but it is uncertain to halt the progression of systemic disease. Reports showed that NNS patients benefit a lot from the treatment of methotrexate (MTX) [11]. Furthermore, it was suggested IFN may be a key mediator of the inflammatory response and may present a therapeutic target in future [9].

## Data Availability

The datasets generated and analyzed during the current study are available from the corresponding author upon request. The c.373C > T and c.355G > A mutations were submitted to ClinVar database [https://www.ncbi.nlm.nih.gov/clinvar/] and accession number were respectively SCV001197254 and SCV001197264.

## Abbreviations

NNS: Nakajo-Nishimura Syndrome
PRAAS: Proteasome-associated autoinflammatory syndrome
JMP: Joint contractures, muscle atrophy, microcytic anemia and panniculitis-induced lipodystrophy
JASL: Japanese autoinflammatory syndrome with lipodystrophy
CANDLE: Chronic atypical neutrophilic dermatosis with lipodystrophy and elevated temperature
LDH: Lactic dehydrogenase
